# Retropseudogene insertion generated through retrotransposition in the *ATP7A* gene results in premature stop codons and a case of Menkes disease

**DOI:** 10.3389/fneur.2025.1680208

**Published:** 2025-11-27

**Authors:** Mengyao Kou, Bin Ren, Mengnan Xing, Lijuan Chen, Xiangping Xu

**Affiliations:** 1 Department of Pediatrics, The First Affiliated Hospital of Harbin Medical University, Harbin, China; 2 Shanghai Nyuen Biotechnology Co., Ltd., Shanghai, China

**Keywords:** Menkes disease, *ATP7A*, retropseudogene, retrotransposon, premature termination codon

## Abstract

Pathogenic variants in the *ATP7A* gene, which encodes a transmembrane copper-transporting P-type ATPase, underlie Menkes disease, a rare X-linked recessive disorder of copper metabolism. We report a 3-year-old boy presenting with progressive neurodegeneration, refractory epilepsy, connective tissue abnormalities, and characteristic kinky hair. Whole-exome sequencing and confirmatory analysis identified a retropseudogene insertion (~500 bp) in exon 3 of *ATP7A*, displaying the hallmarks of target-primed reverse transcription. PCR and RNA-seq revealed a marked reduction in *ATP7A* transcript levels in the patient. This case underscores the diagnostic relevance of retropseudogene insertions in disease genes and highlights their role in human pathology.

## Introduction

Menkes disease (MD, OMIM #309400) is a fatal multisystem disorder of copper metabolism, primarily caused by intragenic variants or partial deletions in the *ATP7A* gene (OMIM #300011). Affected individuals typically present with early-onset neurological deficits, intractable epilepsy, connective tissue dysfunction, skeletal abnormalities, distinctive kinky hair, and urological complications due to loss of *ATP7A* gene function (1). Reported prevalence varies from 1 in 354,507 to 1 in 40,000 based on clinically confirmed cases, while predictive estimates suggest a frequency as high as 1 in 8,664 live male births. Most patients with the classic MD phenotype succumb in early childhood, often from vascular complications or recurrent respiratory infections.

The *ATP7A* gene, located on chromosome Xq13.3, comprises 23 exons and encodes a 1,500-amino-acid transmembrane copper-transporting ATPase (transcript NM_000052.7). Inactivation of *ATP7A* impairs intestinal copper absorption, systemic copper distribution, and the activity of copper-dependent enzymes, including cytochrome C oxidase and dopamine β-hydroxylase (2). Approximately 80% of pathogenic *ATP7A* variants are point mutations, while copy number alterations involving the entire gene account for the remaining 20% (3). Nevertheless, more than half of the identified variants have been classified as variants of uncertain significance (4), highlighting the need to explore additional molecular mechanisms underlying MD.

Transposable elements (TEs), a diverse class of mobile DNA elements, constitute nearly two-thirds of the human genome and play crucial roles in genome evolution and gene regulation (5). Through embedded regulatory sequences such as promoters, enhancers, and open reading frames, TEs can modulate protein-coding gene expression (6). However, TE activity may also be deleterious. Insertions into exonic or noncoding regions can disrupt coding sequences, alter RNA splicing, or cause deletions, thereby producing frameshifts and loss of function (LoF) (7). To date, more than 120 TE-mediated insertions have been implicated in human diseases, including hemophilia, Dent’s disease, neurofibromatosis, and various cancers (8). Retrotransposons, in particular, propagate via a “copy-and-paste” mechanism, creating additional insertions across the genome.

In this report, we describe a retropseudogene insertion generated through retrotransposition in exon 3 of ATP7A, identified in a proband presenting with the classic MD phenotype. Sequence analysis revealed that the insertion introduced multiple premature stop codons, while PCR and RNA-seq confirmed markedly reduced *ATP7A* transcript expression. This case expands the mutational spectrum of *ATP7A* and emphasizes the role of retropseudogene insertions in human genetic disease.

## Materials and methods

### Patient

The proband was a 3-year-old male, born to non-consanguineous parents after an uncomplicated pregnancy and delivery, with no history of perinatal asphyxia. Developmental regression was first noted at 3 months of age, followed by global developmental delay and medically refractory seizures (10–20 per day) that were unresponsive to antiepileptic drugs. As the disease progressed, both seizure frequency and seizure types increased. Electroencephalography (EEG) revealed epileptic spasms, tonic spasms, and focal seizures of mixed types (Supplementary Figure 1).

In addition to neurological symptoms, the patient developed urological complications, including multiple bladder diverticula and recurrent urinary tract infections. Physical examination demonstrated hypopigmented skin, fragile kinky hair (Supplementary Figure 2), skin and joint laxity, a high and narrow palatal vault, and generalized hypotonia. Laboratory testing showed reduced serum copper and ceruloplasmin levels (2.0 μmol/L and 50 mg/L, respectively; normal ranges:11.0–23.6 μmol/L and 200–600 mg/L).

**Figure 1:**
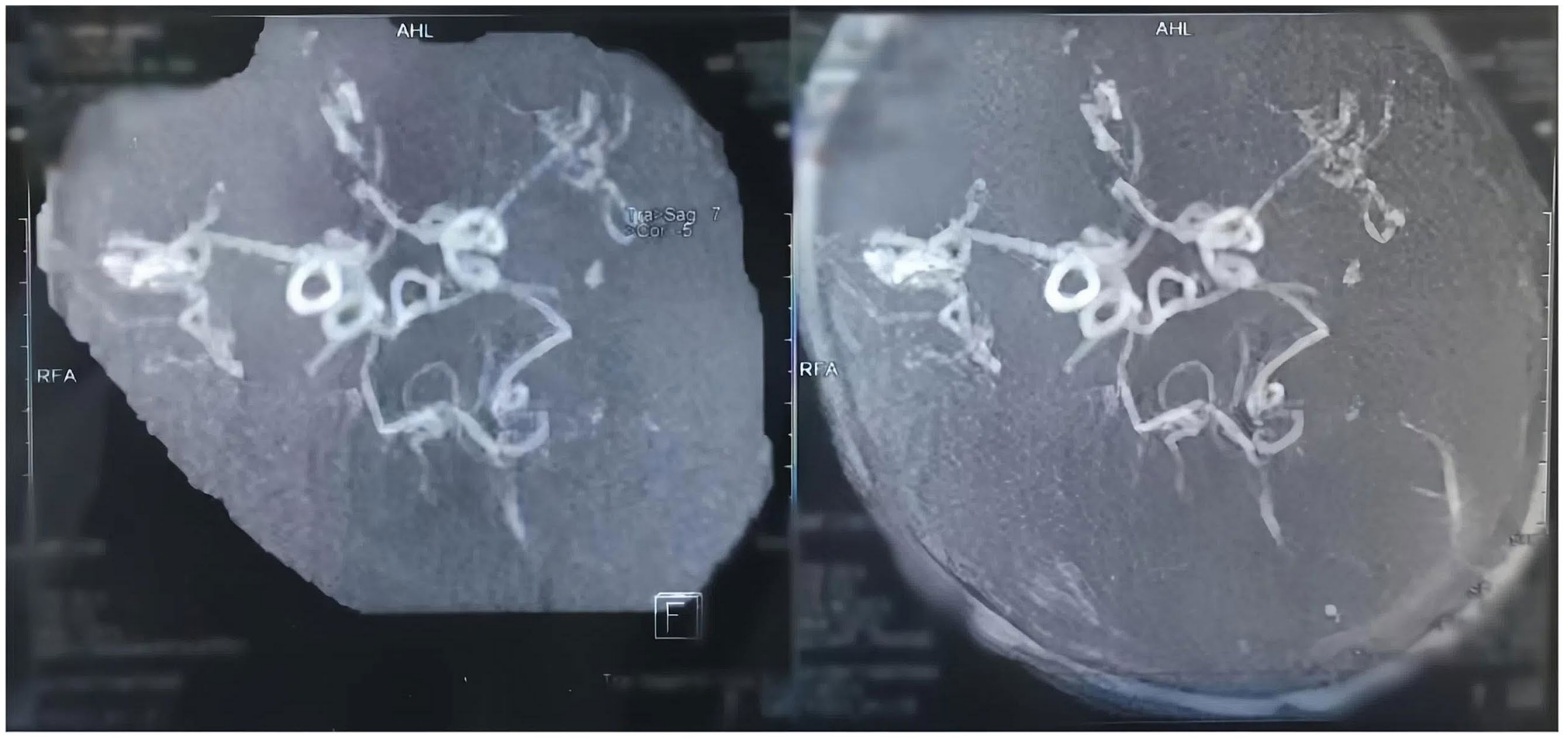
Magnetic resonance angiography results of the proband. The proband’s MRA revealed that the intracranial blood vessels were highly coiled, resembling a screw cone.

Brain magnetic resonance imaging (MRI) demonstrated delayed white matter myelination and diffuse symmetrical cerebral atrophy, while magnetic resonance angiography (MRA) revealed tortuous cerebral vessels (Figure 1). Copper-histidine therapy (0.5 mL daily) was initiated at 11 months of age. Following treatment, serum copper (20.7 μmol/L) and ceruloplasmin (320 mg/L) normalized. Despite biochemical correction, neurological deterioration and seizures persisted, with only partial improvement in hair pigmentation and texture. At the age of 3 years, the patient remained unable to raise his head and exhibited profound deficits in gaze fixation, rolling over, and chewing.

### Molecular genetic testings

After obtaining written informed consent from the patient’s guardian, peripheral blood samples were collected from the proband, who was clinically diagnosed with Menkes disease, as well as from his first- and second-degree relatives. A comprehensive panel of molecular genetic analyses was performed, including whole-exome sequencing (WES), Sanger sequencing, copy number variation (CNV) analysis, multiplex ligation-dependent probe amplification (MLPA), Manta structural variant analysis, chromosome karyotyping, targeted sequence analysis, polymerase chain reaction (PCR), and RNA sequencing (RNA-seq). These stepwise investigations allowed us to elucidate the molecular genetic etiology of the disease and to provide accurate genetic counseling for the family (9).

### Results

WES identified a missense variant in *ATP7A* (c.4390A > G, p. Ile1464Val) in the proband. According to ACMG-AMP guidelines, this variant was classified as benign/likely benign. It has been reported as a polymorphism in the Japanese population (10), and the proband’s family segregation analysis excluded its pathogenicity (Supplementary Figure 3). Structural variant analysis using Manta revealed two chromosomal breakpoints on chromosomes 22 and X, the latter where *ATP7A* is located. However, no deletions, duplications, or balanced chromosomal translocations were detected by CNV analysis, MLPA, or karyotyping. Given the X-linked recessive inheritance of MD and the limitations of detecting noncoding variants, we performed deep targeted WES and transposable element (TE) analysis to further investigate potential pathogenic variants.

**Figure 2:**
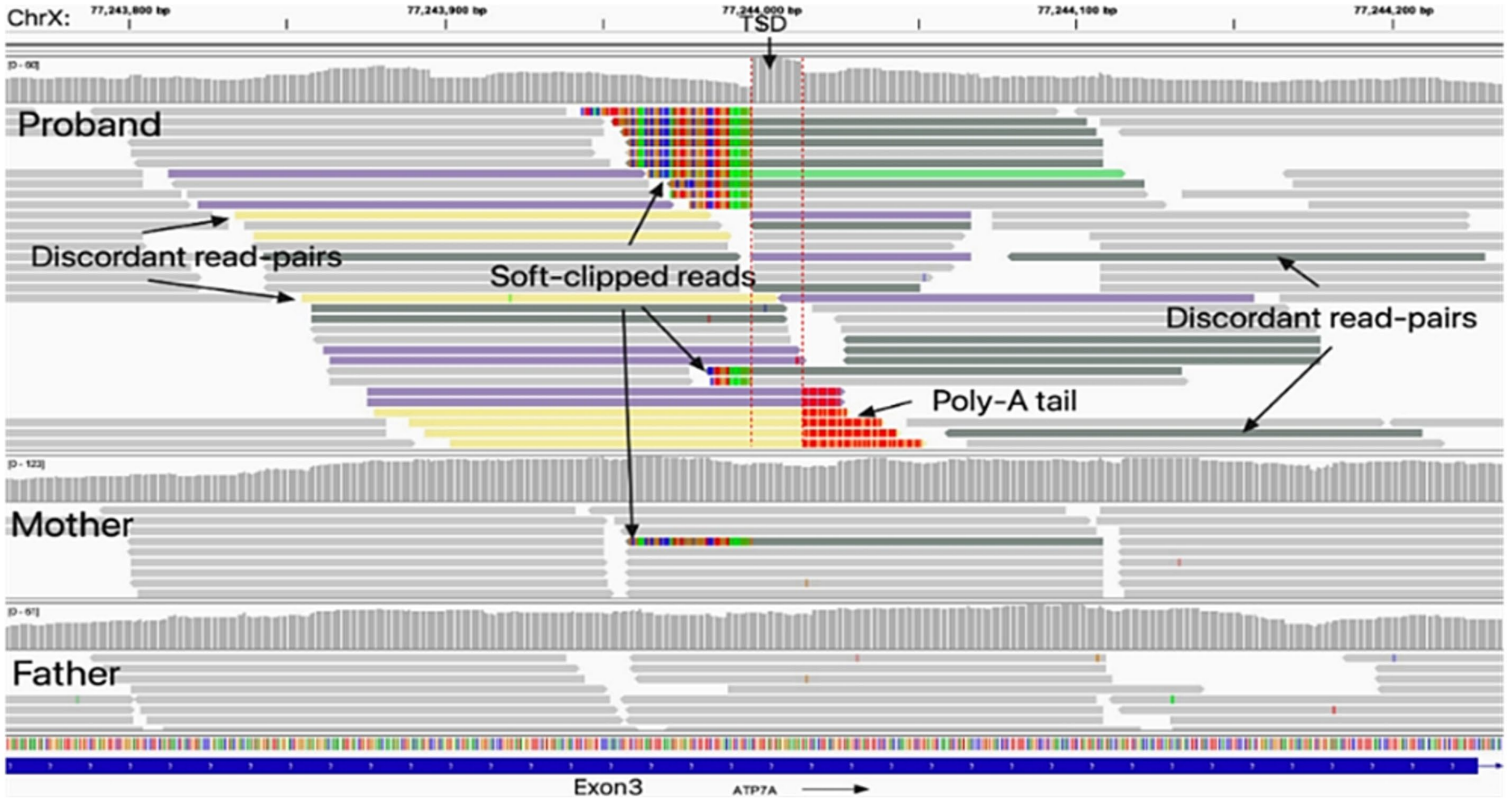
Identification of the insertion generated through retrotransposition in *ATP7A* exon 3. Integrative Genomics Viewer (IGV) analysis revealed an insertion generated through retrotransposition within exon 3 of the *ATP7A* gene in the proband, which was absent in his father; the mother exhibited mosaicism. The non-reference insertion was supported by soft-clipped reads (colored bases) spanning the insertion breakpoints (red dashed lines). Hallmarks of target-primed reverse transcription (TPRT)–mediated retrotransposition were evident, including a target site duplication (TSD) and a poly(A) tail. A sharp decrease in coverage depth was observed at the junction of the insertion.

**Figure 3:**
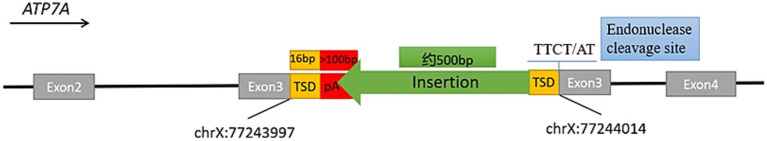
A schematic diagram of the insertion in exon 3 of the *ATP7A* gene. The schematic diagram illustrates the fully resolved exonic insertion at single-base resolution. The ~500 bp insertion generated through retrotransposition contained a 16-bp target site duplication (TSD), a poly(A) tail exceeding 100 bp, and a LINE-1 endonuclease cleavage motif (5′–TTCT/AT–3′).

TE analysis revealed an insertion of approximately 500 bp within exon 3 of *ATP7A* in the proband (Supplementary Figure 4). Further in silico review with Integrative Genomics Viewer (IGV) revealed multiple hallmarks of target-primed reverse transcription (TPRT)-mediated retrotransposition, including soft-clipped reads, poly(A) tails, and a flanking target site duplication (TSD) (Figure 2). Targeted sequencing of junction amplicons resolved the insertion at single-nucleotide resolution, demonstrating a full-length retrotransposon with an uninterrupted poly(A) tail of at least 100 bp at the 3′ end and a 16 bp TSD (GACAATAATCCCTTCT) flanking the site (Figure 3). These findings confirmed that the insertion occurred via TPRT in the reverse orientation. In silico reading-frame analysis further indicated that the insertion introduced multiple premature stop codons, truncating the *ATP7A* transcript and abolishing its functional capacity.

**Figure 4:**
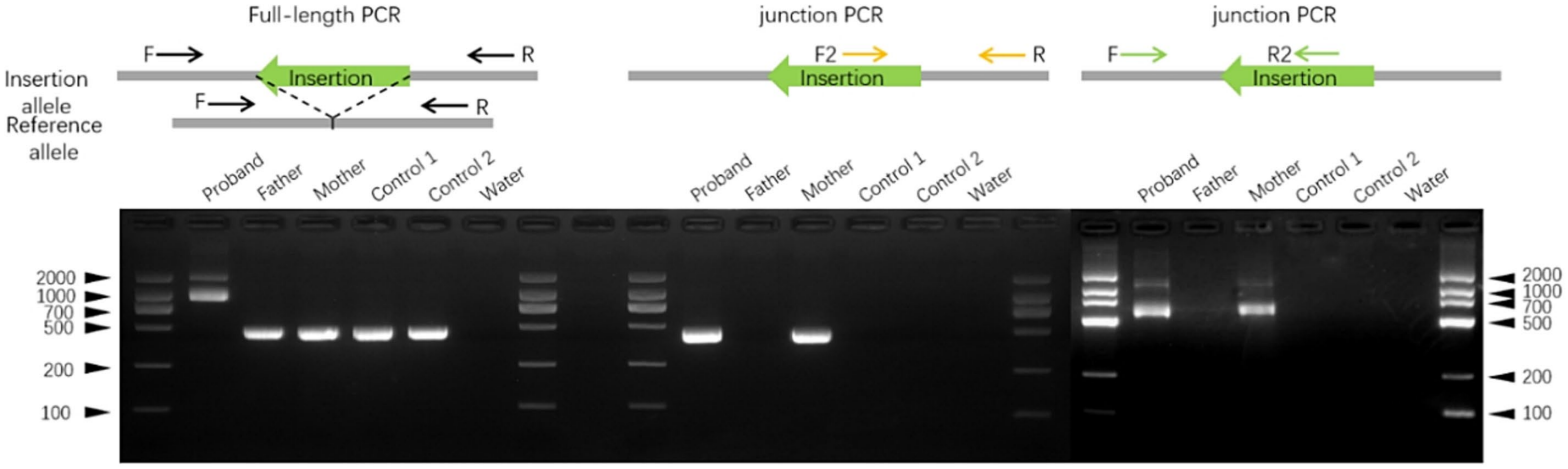
Validation of the insertion in *ATP7A* exon three by PCR. Representative gel electrophoresis images from full-length PCR and PCR using primers flanking both ends of the transposable element confirmed the exonic insertion. Genomic DNA (gDNA) was extracted from blood samples of the proband, his parents, and two unrelated controls.

To confirm the insertion, full-length PCR was performed using primers flanking the affected region. An additional ~500 bp product was observed in the proband compared with DNA from unaffected donors. PCR with primers spanning upstream and downstream regions of the insertion further validated the event (Figure 4).

Finally, we examined the effect of the insertion on *ATP7A* expression using RNA sequencing of peripheral blood. After alignment with STAR and normalization using RPKM, we observed comparable expression levels of *ATP7A* in both parents, whereas the proband exhibited markedly reduced transcript abundance. This reduction in expression is consistent with the predicted pathogenic mechanism and correlates with the clinical phenotype of Menkes disease.

## Discussion

Retrotransposons can profoundly influence chromosome integrity and gene expression, thereby contributing to hereditary disorders (11, 12). Their transpositions can disrupt genomic structure and function, and more than 150 *de novo* retrotransposition events have been reported to date (13). However, these mobile element insertions (MEIs) are often larger than the read lengths of short-read sequencing platforms and are therefore frequently missed by routine genetic testing methods (14). For example, a recent long-read sequencing study identified a 2.8 kb SVA retrotransposon insertion deep within an intron of *ATP7A* in a 16-year-old boy with occipital horn syndrome (OHS) (15). Similarly, exome and Sanger sequencing have been used to characterize an SVA-F retrotransposon in *SMN1* intron 7 as a novel mutational cause of spinal muscular atrophy (16), and PCR combined with Sanger sequencing identified an SVA retrotransposon insertion in *ITGB3* in a family with Glanzmann thrombasthenia (17).

In the present study, we identified a novel insertion in exon 3 of *ATP7A* in a patient with Menkes disease. Sequence analysis demonstrated that the insertion carried hallmarks of LINE-1–mediated retrotransposition, including a 16-bp TSD, a poly(A) tail exceeding 100 bp, and a LINE-1 endonuclease cleavage motif (5′–TTCT/AT–3′). PCR confirmed the insertion, and RNA-seq revealed significant downregulation of *ATP7A* expression in the proband. We hypothesize that the insertion introduced a strong cryptic donor splice site, leading to aberrant splicing and degradation of the transcript via nonsense-mediated decay (NMD). The generation of premature termination codons (PTCs) is consistent with the observed depletion of *ATP7A* expression and provides a plausible molecular mechanism (18).

LINE-1 is the only autonomous transposable element currently active in humans, capable of mobilizing not only itself but also non-autonomous elements such as Alu, SVA, and occasionally cellular RNAs to form retropseudogenes (19). Some processed mRNAs are reverse transcribed and integrated into a staggered chromosome break by the enzymatic machinery of LINE-1 non-LTR retrotransposon, called retropseudogenes (20, 21). Although most retropseudogenes likely represent dead-on-arrival gene copies, they can still influence the evolution and function of genes. These retrotranspositions utilize LINE–1–encoded proteins to relocate to new genomic locations via a coupled reverse-transcription integration mechanism, referred to as TPRT (22, 23). Hallmarks of such events include remnants of poly(A) tails and the presence of TSDs (19). Although many retropseudogenes are transcriptionally inactive, LINE-1-mediated retrotransposition events have long been recognized as drivers of genomic instability, particularly when they occur within coding or regulatory regions. In this case, BLAST and RepeatMasker analyses revealed that this retrotransposon is neither an SVA nor an Alu, but rather a retropseudogene. Sequence alignment demonstrated that the retropseudogene shares 97.7% sequence similarity with a region on chromosome 22, allowing the source of the retrocopy to be traced. The proband’s mother was identified as a mosaic carrier, with amplicon sequencing confirming ~2% chimerism, whereas the father harbored no variants. Notably, the mother’s mosaic status suggests the retrotransposition event occurred during gametogenesis or early embryogenesis, after which it was transmitted in a mosaic state. Functionally, this retropseudogene localizes to exon 3 of ATP7A, which encodes the metal-binding domains (MBDs) harboring the copper-binding GMXCXXC motifs essential for protein function (24). Disruption of this domain likely results in a truncated, non-functional protein.

In conclusion, our findings expand the mutational spectrum of *ATP7A* by identifying a novel retrotransposition-derived, exon-disrupting retropseudogene insertion as a pathogenic cause of Menkes disease. This case highlights the diagnostic challenges of detecting mobile element insertions and underscores the importance of considering such events in unresolved genetic etiologies. Advances in bioinformatics pipelines and re-analysis strategies, particularly those incorporating long-read sequencing, will be essential for improving the detection of MEIs in hereditary disorders.

## Data Availability

The original contributions presented in the study are publicly available. This data can be found here: https://www.ncbi.nlm.nih.gov/bioproject/PRJNA1330472.
