# Supplementary material for: Retropseudogene insertion generated through retrotransposition in the ATP7A gene results in premature stop codons and a case of Menkes disease
Source: Front Neurol. 2025 Nov 27;16:1680208. doi: 10.3389/fneur.2025.1680208 (PMC12696343; doi:10.3389/fneur.2025.1680208)
Supplement: Supplementary file 1 [file Supplementary_file_1.docx]

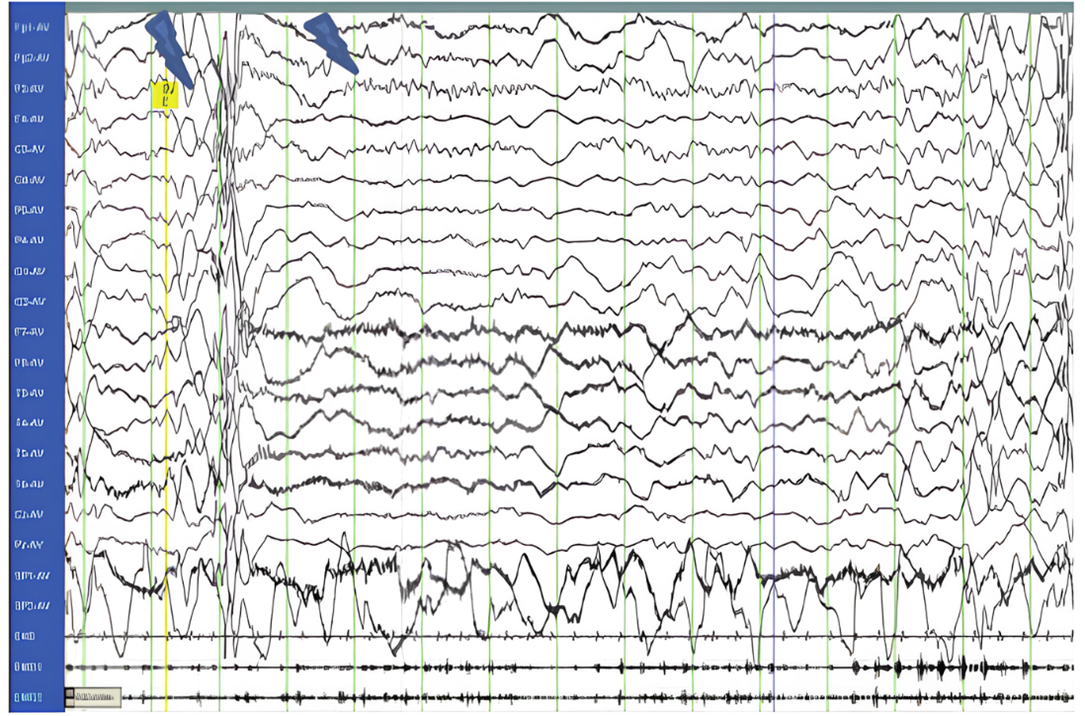


**Online Supplementary Fig.1. The EEG results of the proband in infancy**. EEG showed focal seizures initiated by fast rhythms in the left frontal and central regions after a spasm attack. During the video synchronization period, the proband showed a cessation of movement and a slight deviation of the head and eye to the right.
