# Supplementary material for: Retropseudogene insertion generated through retrotransposition in the ATP7A gene results in premature stop codons and a case of Menkes disease
Source: Front Neurol. 2025 Nov 27;16:1680208. doi: 10.3389/fneur.2025.1680208 (PMC12696343; doi:10.3389/fneur.2025.1680208)
Supplement: Supplementary file 2 [file Supplementary_file_2.docx]

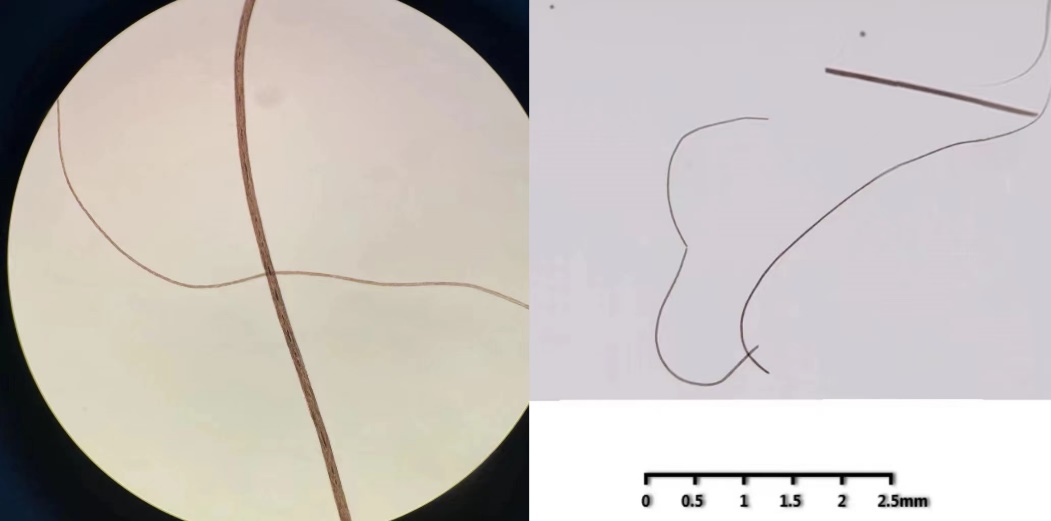


**Online Supplementary Fig.2. The comparison results between the hair of the proband ( before treatment ) and the hair of normal people under the light microscope**. Compared to normal human hair, the proband's hair is thinner, twisted, and easily folds.
