# Supplementary material for: Retropseudogene insertion generated through retrotransposition in the ATP7A gene results in premature stop codons and a case of Menkes disease
Source: Front Neurol. 2025 Nov 27;16:1680208. doi: 10.3389/fneur.2025.1680208 (PMC12696343; doi:10.3389/fneur.2025.1680208)
Supplement: Supplementary file 3 [file Supplementary_file_3.docx]

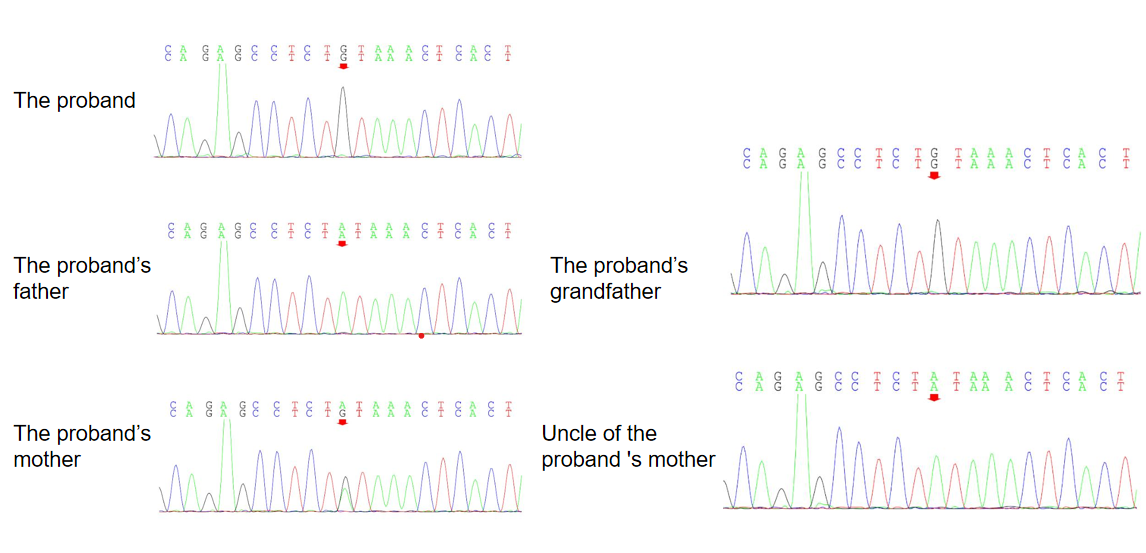


**Online Supplementary Fig.3. Segregation analysis of the proband's family** Sanger sequencing confirmed that the variant site of the proband was derived from his mother and grandfather. However, his grandfather was normal, except for the pathogenicity of the site.
