# Supplementary material for: Retropseudogene insertion generated through retrotransposition in the ATP7A gene results in premature stop codons and a case of Menkes disease
Source: Front Neurol. 2025 Nov 27;16:1680208. doi: 10.3389/fneur.2025.1680208 (PMC12696343; doi:10.3389/fneur.2025.1680208)
Supplement: Supplementary file 4 [file Supplementary_file_4.docx]

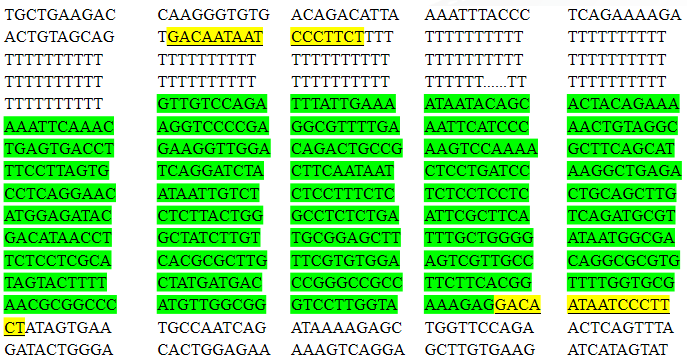


**Online Supplementary Fig.4. The complete sequence of the insertion.** Sequence analysis indicated the presence of a retropseudogene insertion generated through retrotransposition, approximately 500 base pairs in length, featuring at least a 100 base pair uninterrupted poly(A) tail at the 3' end and 16 base pairs of target site duplication (TSD) (highlighted in yellow) on both sides of the insertion site. The inserted segment (highlighted in green)can be compared to the sequence on chromosome 22.
